# Effect of Chitin Whiskers on the Molecular Dynamics of Carrageenan-Based Nanocomposites

**DOI:** 10.3390/polym11061083

**Published:** 2019-06-25

**Authors:** Marta Carsi, Maria J. Sanchis, Clara M. Gómez, Sol Rodriguez, Fernando G. Torres

**Affiliations:** 1 Department of Applied Thermodynamics, Instituto de Automática e Informática Industrial, Universitat Politècnica de Valencia, 46022 Valencia, Spain; mcarsi@ter.upv.es; 2 Department of Applied Thermodynamics, Instituto de Tecnología Eléctrica, Universitat Politècnica de València, 46022 Valencia, Spain; 3 Departament de Química Física, Institut de Ciència dels Materials, Universitat de València, 46010 Valencia, Spain; clara.gomez@uv.es; 4 Department of Mechanical Engineering, Pontificia Universidad Católica del Peru, Lima 32, Peru; sol.rodriguez@pucp.pe (S.R.); fgtorres@pucp.pe (F.G.T.)

**Keywords:** carrageenan, chitin, dielectric relaxation spectroscopy, electric modulus, fragility

## Abstract

Films of carrageenan (KC) and glycerol (g) with different contents of chitin nanowhiskers (CHW) were prepared by a solution casting process. The molecular dynamics of pure carrageenan (KC), carrageenan/glycerol (KCg) and KCg with different quantities of CHWs as a filler was studied using dielectric relaxation spectroscopy. The analysis of the CHW effect on the molecular mobility at the glass transition, *T_g_*, indicates that non-attractive intermolecular interactions between KCg and CHW occur. The fragility index increased upon CHW incorporation, due to a reduction in the polymer chains mobility produced by the CHW confinement of the KCg network. The apparent activation energy associated with the relaxation dynamics of the chains at *T_g_* slightly increased with the CHW content. The filler nature effect, CHW or montmorillonite (MMT), on the dynamic mobility of the composites was analyzed by comparing the dynamic behavior of both carrageenan-based composites (KCg/*x*CHW, KCg/*x*MMT).

## 1. Introduction

Biopolymers have received increasing attention from the scientific community in recent years as a readily available alternative to synthetic polymers. They are obtained from different natural sources and offer a great opportunity to develop new biomaterials with tailored properties for a wide range of applications [1]. Starch, cellulose, alginates, carrageenan, chitin, and different kinds of polysaccharides segregated by plants, algae, and animals are some examples of biopolymers that can be obtained from renewable resources [2,3].

In the last decade, the development of green materials, which intend to reduce human impact on the environment, has attracted the interest of many researchers [4]. In this regard, the development of nanobiocomposites, namely a dispersion of nano-sized fillers into a biopolymer matrix, represents one of the most promising technological advances. Nanocomposites are new materials based on biopolymers and natural fillers which have nanosize. These materials have big potential for applications in the automotive, food packaging and biomedical industries [5,6,7,8,9]. Usually, the addition of a filler leads to a significant change in the matrix properties such as thermal stability, mechanical and barrier properties [10,11].

One of the biopolymers studied over recent years for the development of novel nanobiocomposites the carrageenan (KC), a linear sulfated polysaccharide extracted from red algae. There are different varieties of carrageenan that differ in the content of 3,6-anhydrogalactose, location, and the number of ester sulfate groups [12,13]. The most common types of carrageenans are kappa (κ), iota (i) and lambda (λ) carrageenan [14]. KC is an amorphous polysaccharide with a characteristic glass transition temperature. However, in the literature, few studies report about the glass transition temperature of this material. DSC is shown not to have enough sensitivity to detect *T_g_* in these systems. [15] Thus, according to Mitsuiki et al. [16], *T_g_* values of carrageenans are difficult to detect due to their small heat capacity change at the glass transition. Picker et al. [17] reported that carrageenans *T_g_* is near to 273 K, measured by means of DSC. Kasapis et al. [18] used rheological tests to determine *T_g_* of kappa carrageenan (~266 K). Carrageenan has been used as a thickening agent in the food industry, as well as a biomaterial for biomedical applications [19] and for electrochemical devices [20]. The main limitations of carrageenan-based materials may be linked to their low water vapor barrier and low strength properties. In order to overcome these limitations, carrageenan-based nanocomposites using organic and non-organic reinforcements have been developed [10,15].

Organic fillers have been proposed as alternative reinforcements for the development of polymer-based composites, instead of inorganic clays and ceramic particles. Organic fillers have similar properties to biopolymers, such as biocompatibility, degradability and low toxicity. Micro- and nano-organic fillers could achieve similar properties to non-organic fillers [21]. Among the available natural fillers, chitin (CH) nano-fillers represent a very attractive option because of their nontoxicity, easy modification, low density, biodegradability, and biocompatibility [4,22,23]. Chitin, a semi-crystalline polysaccharide, consisting of 2-acetamido-2-deoxy-D-glucose via a β linkage, is widely distributed in the animal (shrimp, crab, tortoise, insect) and vegetal (fungi, algae) kingdom constituting an important renewable resource. Native chitin occurs as three allomorphs, that is, the α, β, and γ forms depending on the resources, being α-Chitin the thermodynamically most stable and abundant form [24,25].

Chitin represents the second most abundant polysaccharide in nature, only surpassed by cellulose. Both biopolymers, chitin and cellulose, present great structural similarity, being the only difference between them that the hydroxyl group of C2 in cellulose is substituted by an acetamide group in chitin [25,26]. The amide groups allow for the formation of hydrogen bonds between chitin chains and adjacent biopolymer or protein molecules [27]. Chitin nanocrystals or chitin whiskers (CHW) can be prepared from chitin by hydrolysis in a strong acid aqueous medium [28,29]. These CHW have been used as nano-fillers for the preparation of new nanobiocomposites [30,31]. Similar to cellulose whiskers, CHW are able to form rigid 3D networks when used as nano-fillers in polymer nanocomposites [25]. In addition to hydrogen bonding, electrostatic interactions have been reported when using CHW. The amide groups of CHW are positively charged due to the protonation by acid hydrolysis, allowing for electrostatic interaction between CHW and anionic polymers [30,32].

Polymer/CHW nanocomposites usually show different physical properties due to: (i) the different morphology of the composite and (ii) different interactions between the two components [33,34]. Furthermore, in the past, chitin whiskers have been used as a reinforcement in polysaccharide composite films (carrageenan-based films and exopolysaccharides films from cyanobacteria) in order to increase the mechanical properties (elastic modulus and tensile strength) and stability in high moisture conditions [9,35]. According to previous investigations [9], the incorporation of chitin whiskers does not modify the matrix structure nor create new chemical bonds. However, it has been suggested that chitin whiskers form a percolating network based on hydrogen bonding forces, and possible interaction between amide groups of chitin and sulfated ester groups of carrageenan could explain the increase in mechanical properties observed in the mechanical tests.

There are several factors that can affect the CHW reinforcing effect in a polymer matrix such as the glass transition temperature of the polymer matrix, the aspect ratio (*l*/*d* ratio) of CHW, the content of CHW, and the nanocomposite processing technique. In addition to that, it has been shown that a slight modification in the molecular structure can significantly improve the physical properties of a polymeric material. When analyzing the changes in the physical properties of polymeric materials, the understanding of their molecular dynamics is a key point. The dynamics and statics of nanocomposite polymers will be greatly affected by the confinement, as well by the existing interactions between polymer matrix -nanofillers and between nanofillers [36]. Analysis of the effect of the interactions existing between the components of composites (polymer–nanofillers) plays a vital role in the development of new materials. Several reports on the analysis of the glass transition temperature of various nanocomposites demonstrate that *T_g_* may increase or decrease with the addition of nanoparticles. This analysis provides a proper means to understand the behavior of the glass transition temperature in these materials; a key descriptor as the value of this parameter plays an important role in the determination of appropriate applications and processing. For this purpose, molecular dynamic studies by dielectric relaxational spectroscopy (DRS) have been widely employed [37]. Dielectric spectroscopy provides information on the dynamic response and full structural relaxation of nanocomposite polymers. Additionally, dielectric spectroscopy allows for a simultaneous measurement of the relaxation spectrum and the most important temperature that characterizes the behavior of these materials, the glass transition temperature. The *T_g_* of nanocomposites can exhibit considerable deviations relative to the equivalent bulk polymer value. In general, *T_g_* decreases as the interface between polymer and nanofiller produces free surfaces, and increases when the mentioned interface yields attractive interactions as a consequence of the well wetted interface.

In the present study, molecular dynamics of carrageenan-based films are studied by DRS by means of the fragility or steepness index *m* and the glass transition temperature *T_g_*. The present study is focused on the analysis of the effect of chitin nanowhiskers (CHW) on the physical properties of carrageenan films. The carrageenan used in this investigation was previously characterized and was classified as a hybrid kappa-iota (κ/i) carrageenan [15]. We have analyzed the molecular dynamic behavior by means of DRS of four types of samples: biodegradable pure carrageenan (KC), carrageenan-glycerol (KCg) and KCg with different contents of CHW as filler (5, 10 and 15 *wt %*), named as KCg/5CHW, KCg/10CHW and KCg/15CHW, as a function of temperature and frequency. In a previous paper, we have reported the chemical, mechanical and morphological characterization of these films [9,15]. In order to assess the effect of the nature of the nano-inclusions inserted in the carrageenan matrix (KCg), we have compared the DRS results reported in this paper with those published recently by our research group [38] for a related system; specifically, carrageenan composites reinforced with Cloisite^®^Na^+^ that is a layered montmorillonite (MMT) nanoclay.

## 2. Materials and Methods

### 2.1. Materials and Preparation of Films

Carrageenan was extracted using a hot-alkaline extraction method, followed by a non-solvent precipitation using 2-propanol, as reported in our previous work [9,15]. Glycerol (g) of analytical grade was used as a plasticizing agent of the polymeric matrix (KCg). Chitin nanowhiskers (CHW) were obtained following the procedure reported by Wu et al. [39].

Carrageenan-based films were prepared at three different concentrations by weight (5 *wt %*, 10 *wt %* and 15 *wt %*) of chitin nanowhiskers (CHW) using a solution intercalation method. Briefly, carrageenan and glycerol (30 *wt %*) were dissolved in a homogenized CHW suspension (at different concentrations). Next, the solution was poured into Petri dishes and dried in an oven at 313 K. Details of the preparation procedure of the chitin whiskers reinforced carrageenan films as well as their chemical and morphological characterization can be found in recent papers reported by the authors [9,15].

### 2.2. Dielectric Relaxation Spectroscopy (DRS) Characterization

Molecular dynamic characterization of the samples was determined by means of a Novocontrol Broadband Dielectric Spectrometer (Hundsagen, Germany) which involved an Alpha analyzer. The temperature varied from 163 to 313 K in steps of 5 K using the temperature control system Novocontrol Quatro cryosystem, with a precision of ±0.1 K during each sweep in frequency. At each temperature, a frequency sweep from 5 × 10^−2^ to 3 × 10^6^ Hz was measured. The samples were dried at 313 K before each measurement, in a vacuum oven until a constant weight was reached to avoid interference of water in the dielectric response. In order to avoid moisture uptake, during all the DRS experiments the samples were under a steady flow of inert N_2_ atmosphere. This temperature range was chosen because it covers the glass transition temperature and avoids other transitions that can experience the matrix with increasing temperature. Molded disc-shaped samples of approximately 10 mm diameter and 0.12 mm thickness were mounted in the dielectric cell between two parallel cylindrical gold-plated electrodes. Accurate measure of sample thickness was determined with a micrometer screw. The dielectric permittivity and the dielectric loss have been recorded for each sample in dependence on temperature and frequency. The experimental uncertainty was less than 5% in all cases.

## 3. Results

**Figure 1:**
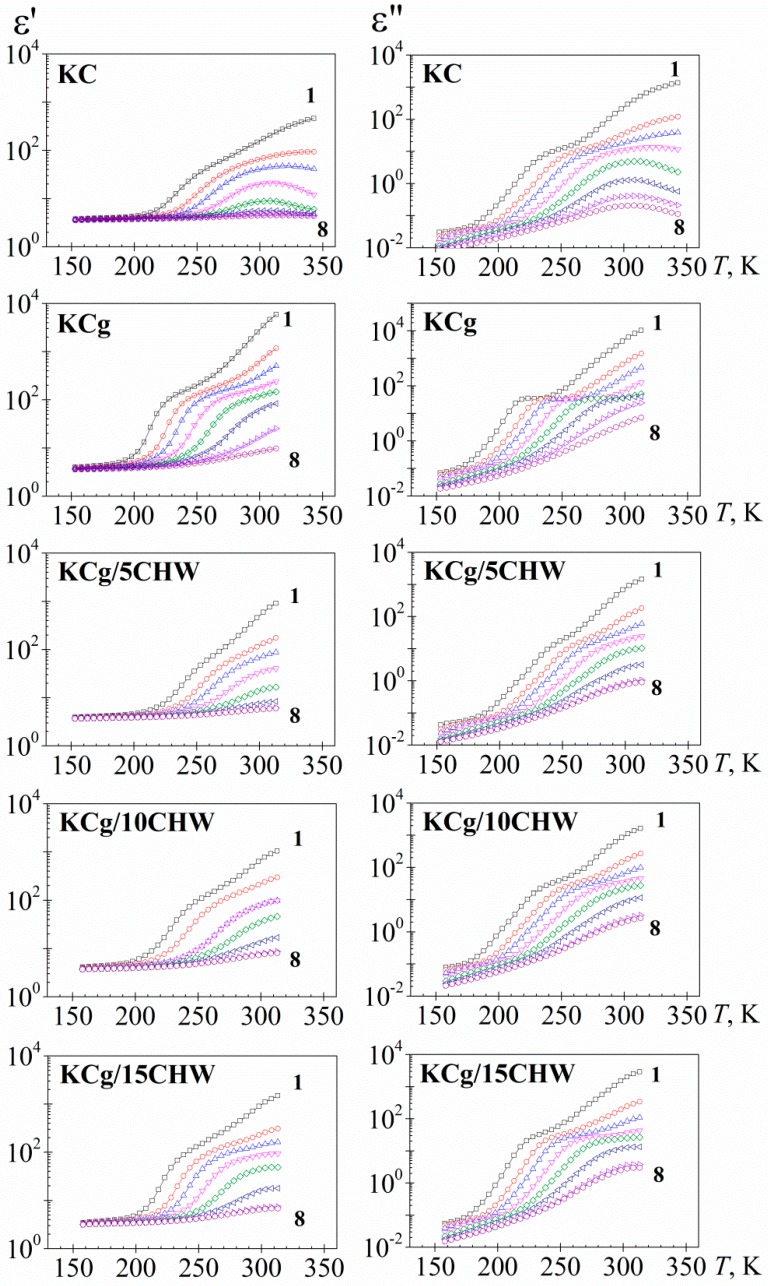
Temperature dependence of the dielectric permittivity, *ε′* (left) and the dielectric loss, *ε″*, (right) for the samples studied, at several frequencies in Hz (**1**: 4.9 10^−2^, **2**: 1.2 10^0^, **3**: 8.7⋅10^0^, **4**: 9.5 10^1^, **5**: 1.0 10^2^, **6**: 1.1 10^4^, **7**: 1.2 10^5^, **8**: 9.1 10^5^).

Figure 1 shows the temperature dependence of the dielectric permittivity (ε′) and dielectric loss (ε″) of the five analyzed samples at eight frequencies. The isochrones of the real component (ε′) of the dielectric complex permittivity (ε*) (figures on the left-hand side) for all samples analyzed displayed a similar pattern; that is, a slight increase of temperature in the low temperature range or glassy state, followed by a steep increase at temperatures located in the vicinity of the glass transition temperature (glass to rubber or α-relaxation), *T_g_*, afterward reaching a pseudoplateau. The isochrones at high temperatures depict a pseudoplateau with a steep increase with increasing temperature is associated with the presence of electrode polarization (EP) phenomena. This effect arises from the charge accumulation in sample-electrode interface [40]. On the other hand, it is necessary to point out that the isochrone of the KC sample, after the glass transition temperature, shows a pseudoplateau with a maximum at around 313 K. This behavior is probably linked to the presence of a structural change experimented by the sample, as has been observed in other materials [41]. In order to avoid such structural change, measurements of the other samples were stopped at 313 K. So, for KCg and KCg/*x*CHW, non-maximum is visualized.

On the other hand, the dielectric loss isochrones (figures on the right-hand side of Figure 1) exhibit, in the intermediate temperature region, a non-well-defined absorption process corresponding to the α-relaxation. By increasing temperature, the loss isochrones of the dielectric permittivity experiment a significant step up due to the presence of conductivity processes related to both ionic conductivity and interfacial polarization effects. The last effects can be originated from the accumulation of charges at the electrode-polymer interface (electrode polarization, EP, process) and from the build-up of charges at the interfaces of the components of the heterogeneous systems (Maxwell-Wagner Sillars, MWS, process) [37]. Due to the experimental temperature range employed in our study, the last interfacial polarization effects are not clearly visible in our spectra. Additionally, fast relaxation absorption (β-relaxation) was observed at lower temperatures. This relaxation is not completely defined in the experimental temperature/frequency range.

**Figure 2:**
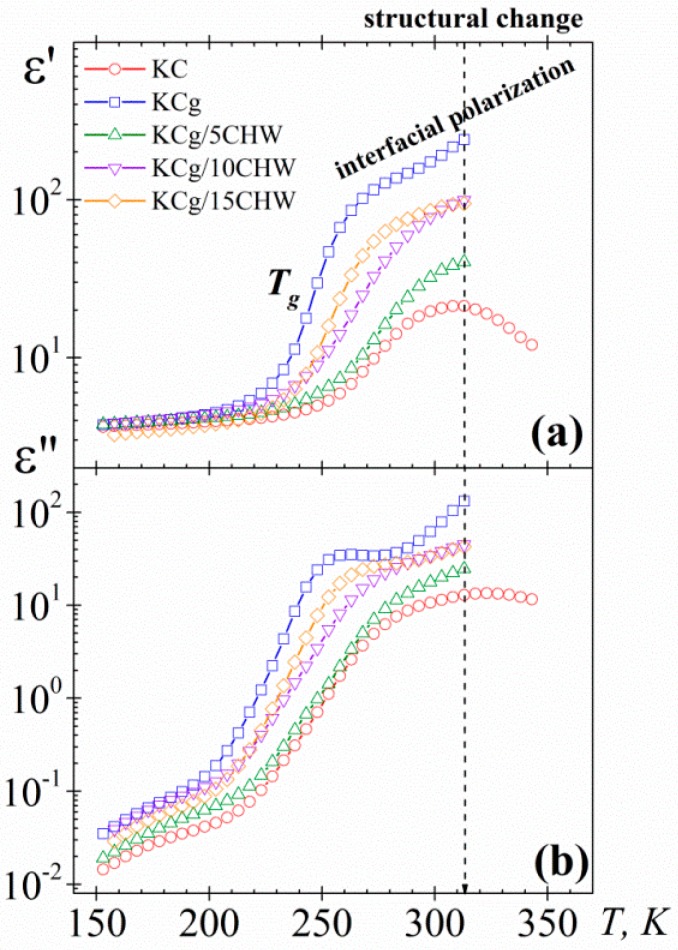
Temperature dependence of (**a**) the dielectric permittivity (*ε′*) and (**b**) the dielectric loss (*ε″*) at 10^2^ Hz for the samples studied.

In order to simplify analysis of the chitin nanowhiskers (CHW) content effect in the dielectric spectra, only the isochrone of 10^2^ Hz for the five samples was plotted in Figure 2. With regard to the dielectric permittivity values, an increase when adding glycerol to the KC matrix was observed. However, the addition of 5% wt of CHW produces a decrease of the dielectric permittivity values. This reduction could be linked to the effect of intercalated CHW within the polymer matrix affecting the regular movement of the long chains of the polymer, and consequently to the dynamic mobility of them. Thus, the dipole orientation may be constrained, making it difficult for the polymer chains to move due to the confinement effects of chitin nanowhiskers [42,43]. Nevertheless, with the addition of higher amounts of CHW (10 and 15 *wt %*), an increasing trend was observed in the permittivity constant, although the values for the composites are lower than those of the matrix.

As mentioned above, according to several authors, the determination of *T_g_* by DSC, for the systems here investigated, has been complicated [15,16,17]. In this regard, DRS has proved to be an outstanding tool for the characterization of the glass transition temperature of different materials. A first estimation of the glass transition temperature (*T_g_*) values trend of the samples (KC and KCg/*x*CHW samples) can be established from the inflection point observed at low temperatures in *ε′* vs. *T* plot, taking into account that this inflection point is related to the change from the glassy state to the glass rubber state. So, according to our experimental results (Figure 2a), at 10^2^ Hz, it is possible to estimate that the *T_g_* of pure KC is about 15 K higher than the *T_g_* of KCg sample. This significant reduction with the addition of glycerol is due to its plasticizing effect. On the other hand, with the addition of a 5% wt of CHW to the KCg matrix an increase of the *T_g_* a value close to the corresponding to KC is observed, due presumably to the mobility restriction imposed by the 5% wt CHW added [15]. However, the addition of higher amounts of CHWs (10% wt and 15% wt) is accompanied by a reduction of *T_g_*, at values that are slightly higher than those of the KCg matrix.

On the other hand, the dielectric loss isochrones of the systems at 10^2^ Hz (Figure 2b) exhibit, at low temperature, two dipolar processes (β and α-relaxations) followed as temperature increases by emerging conductive processes. In all spectra, the α-relaxation, related to the glass transition temperature, is masked by conductive processes, making it difficult to define the maximum of the α-relaxation and complicating the relaxation characterization. The trend observed for the loss permittivity values was the same that the one observed for the permittivity constant.

**Figure 3:**
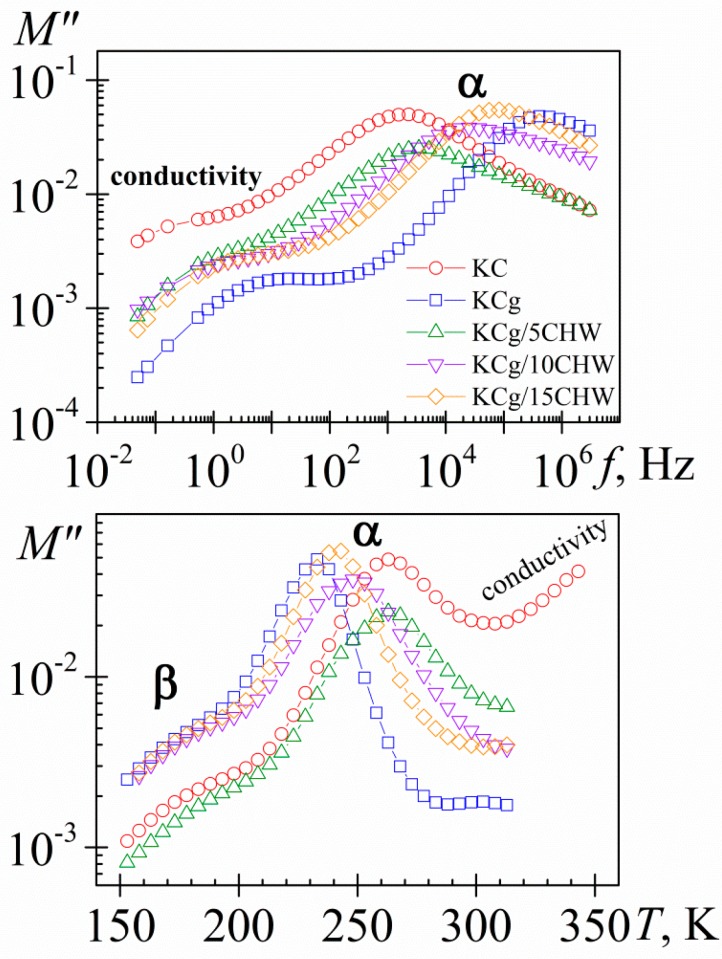
Frequency (**a**) and temperature (**b**) dependence of the loss modulus (*M″*) at 293 K and 10^2^ Hz, respectively. KC (circle), KCg (square) and KCg/*x*CHW nanocomposites with *x* = 5 (up triangle), 10 (down triangle) and 15 (diamond).

To better understand the dipolar and conductive processes taking place in the samples, different plots of the dielectric properties of the materials are employed. One of them is the plot of complex electric modulus, linked to the complex permittivity as M*(ω) = 1/ε*(ω). This representation is very attention-grabbing because it allows us (i) to highlight and characterize the charge transport in the material, (ii) to better visualize the dipolar relaxations features and (iii) to found correlations with the mechanical modulus [44]. In order to simplify the description of the loss modulus spectra, we have plotted for the five samples, the frequency (Figure 3a) and temperature (Figure 3b) dependence of the loss modulus (*M″*) for the isotherm at 293 K and for the isochrone at 10^2^ Hz, respectively. As we can see, the dipolar processes, β- and α-relaxations, are more clearly defined in *M″* plots than in the corresponding of *ε″*. Thus, steady increase in the isochrones *ε″* (Figure 2b) at high temperature is converted into a well-defined peak in *M″* representation (Figure 3b). It is necessary to point out that dipolar relaxation peaks (*M″*) (Figure 3b) shift to lower temperatures with respect to permittivity representation (*ε″*) (Figure 2b), as it would be expected, [(*ε″*/*M″*) = (ε_0_/ε_∞_), where ε_0_ and ε_∞_ denote the unrelaxed and relaxed parts of the ε′, respectively].

In Figure 3, only a slight increase in the broadness of the α-relaxation process was observed by increasing the CHW content, unlike the more significant broadness increase observed by increasing the filler content for the KCg/*x*MMT nanocomposites, reported by us recently [35]. The increased broadness tendency is related to the fact that polymer chain mobility near filler is different from the mobility of the pure polymer. Accordingly, the broadness increase is linked with the increase in heterogeneity. As it would then be discussed, the broadness difference between both types of nanocomposites is related directly to the different interface nature present in both matrix-filler systems.

As in previous related materials [38], because the α-relaxation process is better defined in the loss modulus spectra, the analysis of the CHW content effect on the molecular dynamics of the KCg/*x*CHW (*x* = 5, 10 and 15) nanocomposites was carried out using the Havriliak-Negami (HN) empirical model [45,46] in terms of the dielectric modulus formalism, M* = 1/ε* [37,47]. This analysis was only performed for those isotherms for which the peak maximum was clearly visible. From this analysis, the temperature dependence of the relaxation times was obtained.

**Figure 4:**
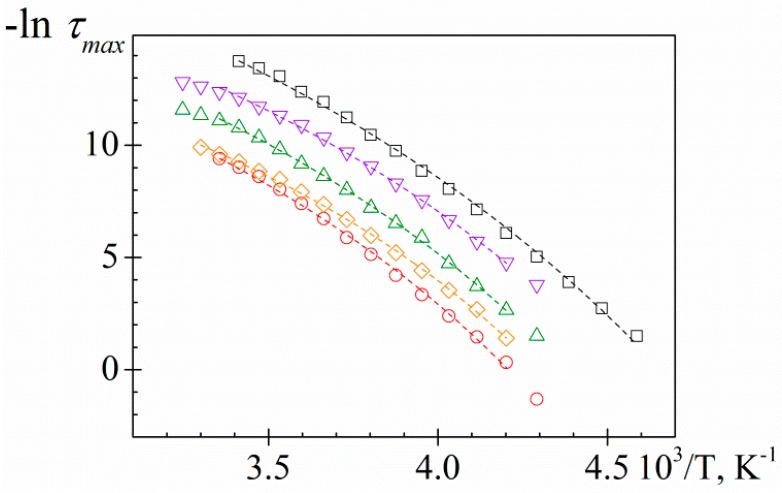
Temperature dependence of the relaxation times for analyzed samples. KC (circle), KCg (square) and KCg/*x*CHW nanocomposites with *x* = 5 (up triangle), 10 (down triangle) and 15 (diamond). Lines are the best fitting to the experimental data.

As usual, the temperature dependence of the relaxation times for the α-relaxation process, related to the glass transition temperature, is parameterized by means of the VFTH equation [48,49,50]:

(1) $$\tau =\tau_{0}\exp\left[\frac{M}{T-T_{v}}\right]$$

where *τ*_0_ is a pre-factor of the order of picoseconds, *M* is a material parameter defining its relaxation activation energy (energetic barrier to molecular rearrangement) and *T_v_*, labeled as Vogel temperature, is the temperature at which *τ* extrapolates to infinity. From the VFTH parameters we can obtain information about the underlying microscopic phenomena, which are directly correlated with macroscopic properties of the material, such as the glass transition temperature, which represents the most important descriptor of amorphous polymers and determines the appropriate type of use and processing. Previously, from the dielectric permittivity spectra (Figure 2a), only an estimation of *T_g_* tendency was carried out. However, from the relaxation map (Figure 4) it is possible to estimate the glass transition temperature, *T_g_*, assuming this is the temperature when *τ_max_* = 100 s [37].

**Figure 5:**
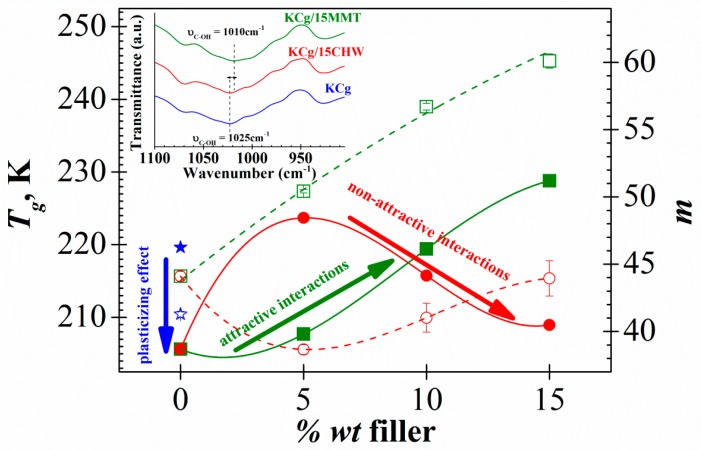
Filler content dependence of the glass transition temperature *T_g_* (full symbols) and fragility index *m* (open symbols) for both KCg/*x*MMT (square) and KCg/*x*CHW (cicle) nanocomposites (*x* = 0, 5, 10 and 15). Stars correspond to the sample without g (KC). The lines only represent the trend behavior. Inset: FTIR spectra of KCg matrix and KCg/15MMT and KCg/15CHW nanocomposites.

The VFTH parameters obtained by the fit of Equation (1) to experimental data and the obtained glass transition temperature, *T_g_*, are summarized in Table 1. Figure 5 shows the dependence of *T_g_* with the CHW content. According to our analysis, the *T_g_* value of pure carrageenan, 219.6 K, decreases about 14.2 K after glycerol addition, as expected by its plasticizing effect. The *T_g_* of KCg/*x*CHW samples is higher than the *T_g_* of KCg. However, the *T_g_* of KCg/*x*CHW samples decreased as the CHW content increased. The *T_g_* tendency obtained is according to that one estimated previously from the inflection point in the *ε′* vs. *T* plot (Figure 2a).

The results of theoretical studies made to investigate the effect of nanoparticles on the glass transition temperature of polymer nanocomposites have shown that *T_g_* can increase or decrease depending of the interaction that occurs within the nanocomposite. In this sense, *T_g_* increase for enough attractive nanofiller surfaces, while it decreases for non-attractive nanofiller surfaces. This fact, together with the estimation that the strength of these interactions is not very strong, make it possible to interpret that the effects associated with non-equilibrium interfacial layers and particle agglomeration, are not dominant [9,36,51,52,53]. However, in some systems this classical tendency is not observed. That is because the dynamics of polymer nanocomposites combine different phenomena, including not only nanofiller–polymer interactions, but also effects related to confinement at larger nanofiller concentration, and interactions between nanofiller and nanofiller networks within the polymer bulk matrix [54]. Accordingly, the *T_g_* depression with the CHW nanofiller content increase could be related to non-attractive polymer-nanofiller interaction. The FT-IR analysis of these nanocomposites reported previously [9] point in the same direction. As shown in the inset of Figure 5, FT-IR spectra of neat carrageenan and carrageenan/CHW films show the same peaks. Namely, the incorporation of chitin does not modify the matrix structure; that is, no chemical interaction between chitin whiskers and carrageenan seems to occur. In addition, heterogeneity in the spatial distribution of the filler and the variation of the degree of crystallinity may be also responsible for the *T_g_* reduction observed. In this sense, reduction in the ultimate tensile strength (UTS) and the elongation at break (EB) values of these nanocomposites reported in our previous paper could also be related to agglomeration issues or non-homogeneous dispersion of CHW [9]. This non-homogeneous distribution with the existence of CHW clusters favors the mobility increase of the polymer chains in the remote areas of them. This should be reflected by a reduction of the glass transition temperature and an increase of the permittivity values up to values close to those of the matrix without reinforcement, as it is observed. On the other hand, the *T_g_* increase observed by adding at 5% wt of CHW to the KCg matrix could be related to the non-local effects of the nanofiller on polymer spatially confinement, that impact the overall relaxation behavior of the composite. This is particularly evident for the case of non-attractive interactions, as the decrease in time near the nanofiller surface and the increase far from the surface can be partially compensated to exhibit not predicted deviations in the overall rate of relaxation [36].

To establish the effect of the nature of the filler in the dynamic mobility of the carrageenan derivate composites, Figure 5 plots the *T_g_* dependence with the filler content of KCg/*x*MMT system studied in a recent paper [38] compared with the actual KCg/*x*CHW system. In this figure, several effects are observed as a function of the type of filler employed, MMT layered or CHW whisker shape. First, the values of *T_g_* rapidly decrease upon addition of glycerol to the carrageenan matrix, whereas the addition of the nanofiller causes opposite trends in both systems. So, the *T_g_* of KCg/xCHW composites shows a maximum at 5 *wt %* meanwhile the KCg/xMMT composites depicts the lower *T_g_* value for this filler content. The addition of higher amounts of filler is accompanied by a reduction or increase in *T_g_* values, depending on the nature of the filler added. These trends can be correlated with the existence of non-attractive KCg/CHW and attractive KCg/MMT interactions found from FTIR characterization for both composite systems [15,38].

**Figure 6:**
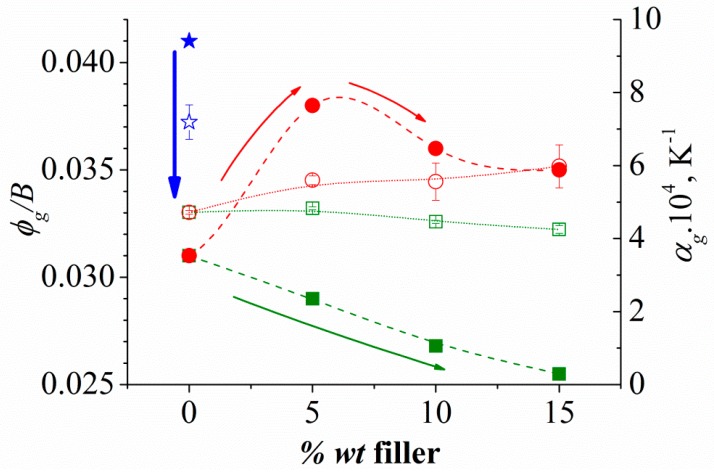
Filler content dependence of the relative free volume at the glass temperature, *ϕ_g_/B* (full symbols) and the value of the expansion coefficient of the free volume, *α_f_* (open symbols) for both KCg/*x*MMT (square) and KCg/*x*CHW (cicle) nanocomposites (*x* = 0, 5, 10 and 15). Stars correspond to the sample without glycerol (KC).

From the VFTH fit parameters, by comparing Equation (1) with the Doolittle expression [55,56], the relative free volume at the glass transition temperature, *ϕ_g_/B* = (*T_g_*–*T_v_*)/*M*, and the expansion coefficient of the free volume, *α_f_* = 1/*M* at *T_g_*, were calculated and included in Table 1. Both *ϕ_g_/B* and *α_f_* values are similar to those found for these parameters for the majority of flexible polymers [57]. Figure 6 plots *ϕ_g_/B* and *α_f_* values as a function of filler content for KCg/*x*CHW and for KCg/*x*MMT [38] to establish an analysis of the filler nature effect. For the KCg/*x*CHW composites, it is evident that by increasing the CHW content, a reduction of the relative free volume at the glass temperature, *ϕ_g_/B*, is obtained, whereas the value of the expansion coefficient of the free volume, *α_f_*, is not practically altered. The same tendency was observed for the KCg/*x*MMT composites. However, for the last system, the numerical values are lower than for KCg/*x*CHW probably due to nanofiller attractive interactions. Non-significant variations in *α_f_* parameter were obtained, for neither of the two composites series, but whereas this parameter decreased slightly with the MMT content, an increase with the CHW content was noticed.

On the other hand, deviation from the Arrhenius behavior can be quantified by the *M* parameter of Equation (1) or by determining the slope at *T_g_* of the curves in the Angell plot [58], leading to the dynamic fragility or steepness index, *m*, which can be evaluated by the following expression:

(2) $$m=\frac{M}{2.303T_{g}{\left(1-T_{v}/T_{g}\right)}^{2}}$$

The *m* parameter has a main role in polymer processing being your evaluation the particular interest for polymeric systems. It provides information of the rapidity with which the properties of the system vary as the temperature of a supercooled liquid approaches its glass transition temperature [59]. Fragile liquids show a significant increase in relaxation times approaching the glass transition than strong liquids. Fragility values typically range between *m* = 16, for strong systems, and *m* = 200 for the fragile ones [60]. The fragility index has been shown to correlate with the non-exponential character of the relaxation function [59,61], the chemical structure of polymers [62,63,64,65], the short-time diffusional properties of supercooled liquids [66,67], nonlinear behavior in the glassy state [68,69,70] and even vibrational motions [71].

The values of the dynamic fragility index, *m*, evaluated from the VFTH fitting parameters according to Equation (2) for all analyzed samples (Table 1) are plotted in Figure 5. In line with our results, the dynamic fragility index slightly decreases with *T_g_*. This trend, that is in not-agreement with Angell’s energy landscape model [72,73], has been observed in other complex polymeric systems [74,75]. The analysis of the composition dependence of the *m* parameter obtained for all analyzed samples reveals that: (i) the addition of glycerol to KC produces a slight increase in *m* value, (ii) the incorporation of 5 *wt %* of CHW filler to KCg matrix results in a decrease in *m* parameter, and (iii) as the CHW content increases the *m* parameter increases yielding a similar value to that of the KCg matrix for the sample with 15% wt of CHW.

To better understand the influence of the filler nature, Figure 5 compares the dynamic fragility index, *m*, corresponding to KCg/*x*CHW and KCg/*x*MMT systems (*x* = 5, 10 and 15). Figure 5 shows that, similar to the behavior of *T_g_*, the value of *m* increases as the filler content increases for composites with attractive polymer-filler interactions (KCg/*x*MMT), whereas both parameters show opposite trends for the composite with non-attractive polymer-filler interactions (KCg/*x*CHW). Besides, for the attractive interaction system (KCg/*x*MMT) dynamic fragility index, *m*, is higher than that of the non-attractive interaction one (KCg/*x*CHW). Both attractive and non-attractive interaction systems show an increase in dynamic fragility index, *m*, with increasing filler content. This trend was evidenced previously in polymer nanocomposites by means of molecular dynamic simulations [36].

**Figure 7:**
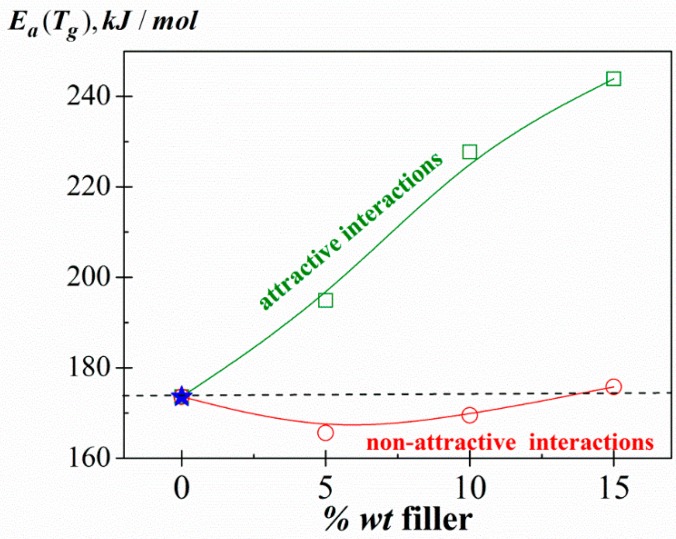
Filler content dependence of the apparent activation energy associated with the relaxation dynamics of the chains at *T_g_*, *E_a_ (T_g_)*, for both KCg/*x*MMT (square) and KCg/*x*CHW (circle) nanocomposites (*x* = 0, 5, 10 and 15). Stars correspond to the sample without g (KC).

Moreover, the apparent activation energy associated with the relaxation dynamics of the chains at *T_g_* was obtained from the dynamic fragility index as $E_{a}(T_{g})=2.303RmT_{g}$ [76]. Figure 7 shows the filler content dependence of the *E_a_ (T_g_)* for both systems, KCg/*x*CHW and KCg/*x*MMT (*x* = 5, 10 and 15). Similar trends were noted for the *m* parameter. As we can see, while non-significant changes in the *E_a_ (T_g_)* values occur when adding increasing amounts of CHW, they increase with the filler content for KCg/*x*MMT nanocomposite series. These trends are in good agreement with the non-attractive and attractive interactions observed in KCg/*x*CHW and KCg/*x*MMT composites, respectively.

## 4. Conclusions

The dynamic molecular analysis of the KCg/*x*CHW composites synthesized by a solution casting process was carried out by using DRS. This technique with a high resolution, has proved to be a very useful tool to investigate the molecular dynamics of KCg/*x*CHW nanocomposites. The glass transition temperature, *T_g_*, of these nanocomposites, a determinant parameter for their processing and applications, has been determined using DRS since it could not be measured by DSC. The effect of the CHW content on the molecular mobility, at the glass transition of the nanocomposites was analyzed using the electric modulus formalism.

As the CHW content increased, the intensity, the broadness and the temperature/frequency of the maximum of the glass rubber transition were modified:

i. By addition of CHW filler, the relaxation process was shifted to lower frequencies, up to two orders of magnitude compared to the unfilled polymer, and to higher temperatures. Like this, the *T_g_* evaluated from DRS measurements increases from 205.4 K (KCg) to 223.7 K with the incorporation of a 5% wt of CHW, but as the CHW content increases, a decrease of the *T_g_* to 209 K for a 15 *wt %* was observed. These trends indicate a decrease and increase, respectively, of molecular mobility by modifying the CHW content.
ii. As the content of CHW filler increased, the height of the peak decreased. Thus, the CHW incorporation restricted the movement of the polymer molecules near the nanowhiskers surface. Consequently, the number of polymer chains that participate in the process was reduced.
iii. The broadness of the α-relaxation increased slightly with the addition of the CHW filler to the KCg matrix. This tendency is related to the fact that polymer chain mobility near CHW is not the same as the mobility of the pure polymer.
iv. A reduction of the fragility index *m* was obtained with the filler CHW addition to the KCg matrix. The lower value was obtained when a 5% of CHW was added to the KCg matrix, increasing the fragility index *m* as the CHW content rose.

Also, a comparative analysis with data previously published for KCg/*x*MMT nanocomposites was carried out. According to our previous results, attractive interactions and non-attractive interactions were presented in KCg/*x*CHW and KCg/*x*MMT nanocomposites, respectively. The nature of these interactions is determinant when studying nanocomposites molecular dynamics. The glass transition temperature and the fragility can be tuned through the judicious choice of the nanofiller. So, this choice has numerous implications in the physical properties of resulting polymer nanocomposites, and therefore in the practical application of them.

## Figures and Tables

**Table 1:** Sample code and weight fraction (%) of chitin nanowhiskers (CHW) content and Vogel-Fulcher-Tammann-Hesse (VFTH) fit parameters, glass transition temperature evaluated from DRS analysis (*τ* = 100s), relative free volume (*ϕ_g_/B*), expansion coefficient (*α_g_*), fragility index (*m*) and activation energy (*E_a_(T_g_)*) associated with the glass-rubber relaxation at *T_g_* of KC and KCg/*x*CHW samples (*x* = 0, 5,10 and 15).

Sample Code	KC	KCg	KCg/5CHW	KCg/10 CHW	KCg/15 CHW
*wt %* CHW	0	0	5	10	15
*τ*_0_ (s)	10^−8.6^ ± 10^0.3^	10^−12.1^ ± 10^0.1^	10^−11.4^ ± 10^0.2^	10^−11.9^ ± 10^1.8^	10^−11.1^ ± 10^1.8^
*M(K)*	1390.6 ± 12.2	2119.3 ± 22.4	1786.0 ± 21.3	1799.8 ± 20.2	672.8 ± 20.3
*T_v_(K)*	162.9 ± 4.6	140.1 ± 0.6	156.7 ± 0.7	151.6 ± 6.1	150.2 ± 6.5
*T_g_^DRS^* (K) (τ = 100 s)	219.6	205.4	223.7	215.7	209.0
*ϕ_g_/B*	0.041 ± 0.002	0.031 ± 0.002	0.038 ± 0.003	0.036 ± 0.003	0.035 ± 0.003
α_g_⋅10^4^, K^−1^	7.19 ± 0.47	4.72 ± 0.09	5.60 ± 0.12	5.56 ± 0.51	5.98 ± 0.59
*m*	41.3 ± 2.2	44.1 ± 0.3	38.7 ± 0.4	41.0 ± 2.7	43.9 ± 3.1
*E_a_(T_g_)*, kJ mol^−1^	173.6 ± 3.4	173.6 ± 3.8	165.6 ± 4.3	169.5 ± 38.9	175.8 ± 46.2
